# Antimicrobial Resistance: Stewardship and One Health in the Eastern Mediterranean Region

**DOI:** 10.7759/cureus.58478

**Published:** 2024-04-17

**Authors:** Abdulqadir J Nashwan, Muna Barakat, Faizan Niaz, Samiuddin Tariq, Sirwan K Ahmed

**Affiliations:** 1 Department of Nursing Education and Research, Hamad Medical Corporation, Doha, QAT; 2 School of Pharmacy, Applied Science Private University, Amman, JOR; 3 Department of Clinical Medicine, Dow Medical College, Dow University of Health Sciences, Karachi, PAK; 4 College of Nursing, University of Raparin, Rania, IRQ

**Keywords:** interdisciplinary collaboration, eastern mediterranean countries, one health approach, antimicrobial stewardship, antimicrobial resistance

## Abstract

Antimicrobial resistance (AMR) is a major threat in the Eastern Mediterranean region (EMR) due to factors such as the high prevalence of infectious diseases, weak health systems, and the misuse of antimicrobials. This paper aims to discuss how interdisciplinary action and collaboration, specifically through antimicrobial stewardship (AMS) and the One Health approach, can effectively address AMR in the EMR. The review focuses on successful AMS initiatives and the adoption of the One Health approach in countries within the EMR, including the Gulf Cooperation Countries (GCC), Egypt, Iran, Jordan, and Pakistan. The goal is to highlight the potential for progress in combating AMR and identify challenges and opportunities for strengthening interdisciplinary collaboration. The results showcase successful AMS programs and One Health initiatives in various EMR countries, demonstrating their potential to address AMR challenges. The paper also discusses the challenges faced by these nations, such as limited resources, fragmented health systems, and knowledge gaps. Additionally, opportunities for enhancing interdisciplinary action through regional cooperation, international partnerships, and research and innovation are outlined. In conclusion, this paper emphasizes the importance of a comprehensive and collaborative response to combat AMR in the EMR. It advocates for the One Health approach as a crucial framework to guide these efforts, promoting coordinated action, improved surveillance, responsible antimicrobial use, and enhanced interdisciplinary collaboration to effectively mitigate the threat of AMR.

## Introduction and background

Antimicrobial resistance (AMR) presents a significant risk to global public health [1]. The misuse and overuse of antimicrobials in human medicine, agriculture, and veterinary practices are primarily responsible for the rise of AMR [2]. To effectively tackle this issue, an interdisciplinary approach is crucial. One Health, a global initiative that recognizes the interconnectedness between human, animal, and environmental health, provides an ideal framework for fostering collaboration in addressing AMR [3]. It brings together knowledge from fields such as human medicine, veterinary medicine, environmental science, and public health. The goal is to understand and reduce complex relationships between humans, animals, and ecosystems. This approach involves working together on research, surveillance, and policy development to promote sustainable practices, prevent disease spread, and protect populations and the environment. Ultimately, One Health strives to improve global health by recognizing and addressing interconnectedness. The concept of One Health acknowledges the dependence on the well-being of humans, animals, and the environment [3]. Understanding the correlation between AMR and One Health is vital in addressing the problem. Microorganisms, such as bacteria, viruses, and fungi, have developed resistance to the effects of antimicrobial drugs, resulting in less effective treatments and an increased risk of disease. The One Health approach recognizes that AMR has various origins in the human, animal, and environmental sectors. In human medicine, the improper use of antibiotics is a primary contributor to the emergence of resistant strains [4]. In veterinary medicine, the extensive use of antimicrobials for livestock growth promotion and disease prevention can lead to the emergence of resistant bacteria in animals and the environment [2]. Moreover, the discharge of antimicrobial residues from agricultural and industrial sources can contaminate water and soil, further promoting resistance [1]. During the coronavirus disease 2019 (COVID-19) pandemic, there has been increased concern about AMR due to several reasons [5]. The use of antibiotics to treat secondary bacterial infections in COVID-19 patients has accelerated the development of AMR, making it more difficult to manage infectious diseases in the future [5]. The widespread prescription of antibiotics, often without appropriate diagnostics, has hastened the development of resistant strains, posing a long-term threat to public health [6]. The disruptions in healthcare systems have also led to inappropriate antibiotic use, exacerbating the problem of AMR. Additionally, the focus on COVID-19 has diverted resources from AMR surveillance and research, hindering efforts to address this global health threat. To effectively combat AMR during and after the pandemic, it is crucial to implement coordinated strategies that prioritize rational antibiotic usage, robust surveillance systems, and ongoing research to preserve the effectiveness of antimicrobial drugs.

This article explores the role of interdisciplinary action and collaboration in addressing antimicrobial resistance, antimicrobial stewardship (AMS), and One Health, with real-life examples from Eastern Mediterranean countries.

## Review

AMR in Eastern Mediterranean countries

*Factors* *Driving AMR and Concerns in the Eastern Mediterranean Region (EMR)*

EMR countries are highly susceptible to AMR due to several factors. The excessive and incorrect use of antibiotics is a major factor in the development of AMR. Antibiotics are often used inappropriately or inaccurately, leading to the emergence of drug-resistant bacterial strains. Inadequate infection control measures in healthcare facilities and insufficient sanitation and hygiene protocols in communities also contribute to the spread of AMR. These factors create an environment that allows antibiotic-resistant bacteria to multiply and spread rapidly, posing a significant risk to public health. In addition, these factors include a high prevalence of infectious diseases, weak health systems, and limited access to high-quality healthcare [7]. Specifically, Egypt, Iran, and Pakistan have experienced the rapid emergence of resistant pathogens due to the inappropriate use of antibiotics and inadequate regulation of antimicrobial drug sales [8-10]. For example, a comprehensive study conducted in Egypt found that a concerning 81% of Escherichia coli isolates displayed resistance to at least one antibiotic, with 62.5% showing resistance to multiple drugs [11]. To facilitate policy development and strategic planning for collaborative efforts, Hasan et al. conducted a review of tuberculosis (TB) control and AMR programs in the EMR in 2016 [12]. This review identified gaps in TB care in several EMR countries, mainly due to limited resources. However, valuable insights from TB control initiatives can help shape emerging AMR programs. To promote cooperation between these two programs, the introduction of a logic model was proposed.

Using a logic model can improve the analysis of gaps in TB control and AMR programs in the EMR. A logic model provides a structured framework for understanding the inputs, actions, outputs, outcomes, and implications of public health interventions. Within the framework of TB control and AMR initiatives, a logic model can identify specific areas that require enhancement and guide decision-making. The logic model can be used to identify the necessary resources and inputs for TB and AMR initiatives, including financial support, healthcare professionals, diagnostic equipment, and medications. By identifying these inputs, stakeholders can prioritize resource allocation and ensure that essential components receive sufficient resources. Additionally, the logic model delineates the actions and interventions carried out within TB and AMR programs, such as diagnostic testing, treatment procedures, surveillance techniques, and public awareness campaigns. This systematic approach enables the assessment of program implementation and the identification of potential obstacles or inefficiencies. Furthermore, the logic model helps define the outcomes of TB and AMR programs, including the number of TB cases detected, rates of successful treatment, compliance with antibiotic stewardship measures, and changes in public awareness and behavior regarding AMR. These outputs serve as intermediate indicators of the program's effectiveness and provide information for ongoing monitoring and adjustments. Moreover, the logic model facilitates the evaluation of various outcomes, such as a reduction in TB incidence and mortality rates, a decrease in the prevalence of drug-resistant TB strains, improvements in antibiotic prescribing practices, and the strengthening of healthcare systems' capacity to address infectious diseases and AMR. The logic model allows stakeholders to monitor the long-term effects of TB and AMR initiatives, including advancements in public health, reduced healthcare expenses, enhanced community capacity to combat infectious diseases, and lower chances of widespread AMR. Incorporating a logic model into TB control and AMR programs in the EMR provides a systematic approach to designing, implementing, tracking, and evaluating interventions. This ultimately enhances the level of analysis and contributes to the effectiveness of public health efforts.

Talaat et al. conducted a study to assess the burden of AMR in the EMR [13]. The study analyzed data from 2017 to 2019 on bloodstream infections reported to the Global Antimicrobial Resistance Surveillance System, national surveys on antimicrobial prescriptions from seven countries, and two regional surveys. The data revealed alarmingly high rates of resistance, particularly for carbapenem-resistant Acinetobacter spp. (with an incidence of 70.3%) and carbapenem-resistant Escherichia coli (with an incidence of 4.6%). The existing deficiencies in infrastructure for infection prevention, control, and AMS in most regional countries demand urgent political intervention at the regional level to address the AMR issue. Okeke et al. conducted a global review emphasizing that AMR is a significant concern worldwide, especially in developing nations with infectious diseases and limited financial resources [14]. Despite the lack of surveillance systems in many developing regions, available data shows increasing resistance rates due to antimicrobial misuse, poor infection control, and public health deficiencies. Both humans and animals can serve as reservoirs of resistance, posing risks to public health and causing economic burdens. In addition, Borg et al. conducted a study on the susceptibility of invasive Escherichia coli isolates in various countries, including Algeria, Cyprus, and Egypt in the EMR, from January 2003 to December 2005 [15]. Results showed that 18.9% of the isolates were resistant to third-generation cephalosporins and 21.0% were resistant to fluoroquinolones. Countries in the EMR, particularly Egypt with a resistance rate of 31%, reported a significant prevalence of multi-resistant strains. These findings highlight the need for further investigation into the causes of resistance and improved surveillance in the EMR. *Migration Impact on AMR*

**Figure 1:**
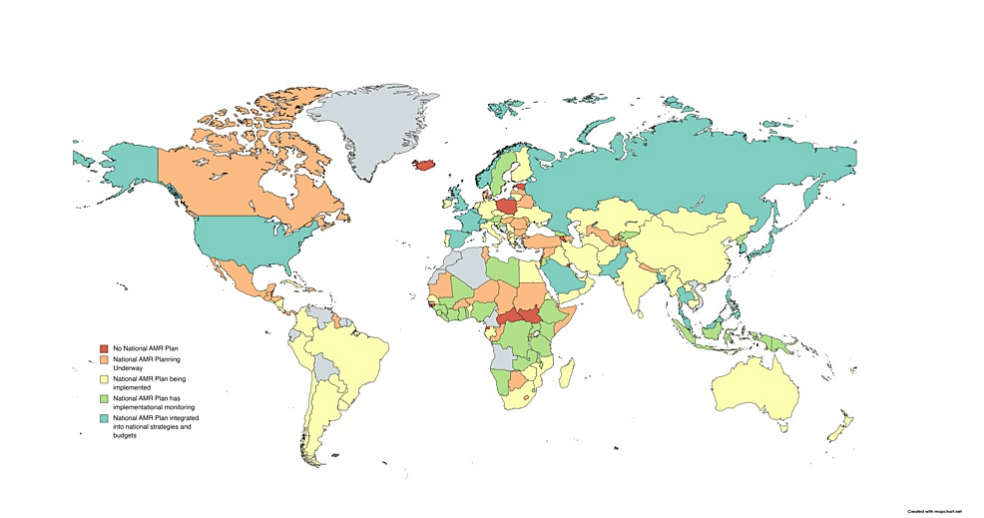
Global overview of antimicrobial resistance (AMR) programs including EMR countries. This figure has been created by Faizan Niaz, a co-author of this article. As reported by the World Health Organization (WHO) at [http://www.amrcountryprogress.org/]. EMR: Eastern Mediterranean Region

Simultaneously, a systematic review and meta-analysis assessed the impact of migration on AMR in Europe [16]. Twenty-three observational studies comprising data from 2319 migrants were evaluated, revealing a pooled prevalence of 25.4% for AMR carriage or infection among European migrants. Significant rates of resistance were observed against Staphylococcus aureus and Gram-negative bacteria. Notably, refugees and asylum seekers in Europe exhibited a higher prevalence (33.0%) of AMR carriage or infection compared to other migrant groups (6.6%) [16]. Methicillin-resistant Staphylococcus aureus (MRSA) was found in varying rates in Europe, Africa, and EMR countries, which are major sources of migrants to Europe. Furthermore, there was a slightly higher prevalence of AMR in settings with high concentrations of European migrants, such as refugee camps and detention facilities, compared to hospitals [16]. Importantly, there is limited evidence of significant transmission of AMR from migrants to host populations. Therefore, it is crucial to improve living conditions and healthcare accessibility for refugees, migrants, and host communities, as well as address the factors contributing to AMR throughout the migration process. Figure 1 provides global data on the status of AMR programs according to the World Health Organization (WHO) [17].

Role of interdisciplinary action and collaboration in AMS

The AMS refers to coordinated interventions aimed at improving and evaluating the appropriate use of antimicrobials [18]. Implementing AMS requires a multidisciplinary approach involving physicians, pharmacists, nurses, microbiologists, veterinarians, and other healthcare professionals. In the Eastern Mediterranean countries, successful AMS initiatives have been achieved through interdisciplinary collaboration.

*AMS Efforts in Jordan* In Jordan, the Ministry of Health recognizes the importance of AMS and has taken measures to promote the proper use of antibiotics. In 2014, the ministry established the National Antimicrobial Resistance Committee, which is responsible for developing national policies and guidelines related to antimicrobial resistance and stewardship [19]. The committee includes experts from various health sectors, such as infectious disease specialists, microbiologists, and pharmacists. Several hospitals in Jordan have also implemented AMS programs to encourage the responsible use of antibiotics. For example, the Jordan University Hospital in Amman has created a dedicated AMS team that reviews antibiotic prescriptions and provides recommendations to healthcare providers. Additionally, the team conducts education and training sessions on AMS for healthcare providers [20]. In addition to these efforts, multiple studies have been conducted in Jordan to assess the prevalence of antimicrobial resistance and the effectiveness of stewardship interventions [21-23]. A recent study in Jordan found that a comprehensive approach, including education and feedback for healthcare providers, successfully reduced the inappropriate use of antibiotics in a Jordanian hospital [20]. However, more work needs to be done to further enhance AMS in Jordan. The government and healthcare providers are taking steps to promote the appropriate use of antibiotics and reduce the risk of antimicrobial resistance [24].

AMS Efforts in Iran

In Iran, a National Action Plan for the Containment of Antimicrobial Resistance was formulated in 2016 [25]. This plan emphasized the importance of interdisciplinary collaboration across diverse sectors, including human health, animal health, agriculture, and the environment. Within this plan, the establishment of an AMR surveillance system, the promotion of rational antimicrobial use, and the improvement of infection prevention and control practices were identified as crucial strategies. *AMS Initiatives in Gulf Cooperation *Countries (*GCC)*

The GCC, including Bahrain, Kuwait, Oman, Qatar, Saudi Arabia, and the United Arab Emirates (UAE), have recognized the significance of AMS in addressing the threat of Antimicrobial Resistance (AMR) [26-29]. In recent years, these countries have implemented various initiatives and programs to promote AMS. In Bahrain, the Ministry of Health has devised a national strategic plan to combat AMR, with a specific focus on AMS. The Bahraini AMS program includes training healthcare professionals on the rational use of antimicrobials and the establishment of an electronic surveillance system to monitor antimicrobial consumption [4]. Kuwait has introduced several AMS programs, such as implementing antibiotic restriction policies and establishing AMS committees in hospitals [30]. In 2019, Kuwait launched the National Action Plan for Antimicrobial Resistance, which emphasizes the need for a collaborative and intersectoral approach to address AMR [29]. Efforts in Oman to implement AMS have made significant progress, with the Ministry of Health developing guidelines for antimicrobial use and establishing a national antibiotic committee to oversee and regulate antimicrobial prescriptions [30]. Qatar has also instituted an AMS program in its largest chain of hospitals, Hamad Medical Corporation, with the primary goal of improving antimicrobial prescribing practices and enhancing infection prevention and control measures [31]. Saudi Arabia has taken steps to establish a National Antimicrobial Stewardship Program, which includes the development of guidelines for antimicrobial prescription, infection prevention and control measures, and the implementation of an antimicrobial consumption surveillance system [32].

Research and Findings Related to AMS

A recent study conducted in Hail, Saudi Arabia by Alanazi et al. revealed that respiratory tract infections were the most common infectious disease among hospital patients, primarily affecting individuals in their 20s [33]. Additionally, gallbladder calculi with cholecystitis accounted for the majority of multiple infections (40.3%). The study found that beta-lactam antibiotics were the most frequently prescribed, followed by fluoroquinolones and macrolides. However, culture sensitivity tests were rarely performed (3.8%). The study emphasizes the need to promote culture sensitivity testing and implement guidelines for AMS to ensure responsible antibiotic use. Another study conducted in the same region by Almansour et al. used a comprehensive questionnaire distributed to 202 physicians [34]. The study found that 43.56% of the physicians believed prescribing behavior contributes to antibiotic resistance. Interestingly, while 49% of respondents prescribed antibiotics daily, only 36.13% frequently discussed antibiotic resistance with their patients, and 6.4% never addressed it at all. Most general practitioners were aware of the factors leading to antibiotic resistance but seldom communicated this to patients, presuming patients lacked understanding. Addressing these prescribing behaviors might be the key to reducing antibiotic resistance. Furthermore, a study conducted at a tertiary care hospital in Al Baha, Saudi Arabia examined children under 14 admitted with urinary tract infections (UTIs) between May 2017 and April 2018 [35]. Of the 118 urinary samples, Escherichia coli was the most frequent causative agent, with a higher infection rate in girls (68.64%) than boys (31.36%). Nitrofurantoin was the most commonly prescribed drug, followed by trimethoprim/sulfamethoxazole and amoxicillin/clavulanic acid. Due to the increasing prevalence of antibiotic resistance, the authors emphasize the importance of routine microbial culture and sensitivity testing from urine samples before initiating treatment, rather than relying solely on standard guidelines. In the UAE, the Dubai Health Authority has initiated an AMS program. This program aims to enhance the prescribing practices of antimicrobials, establish treatment protocols for antimicrobial use, and raise awareness and knowledge among healthcare professionals [36,37]. These efforts across the GCC nations exemplify their dedication to AMS and offer beneficial illustrations of the efficacy of interdisciplinary cooperation in tackling AMR.

Adoption of the One Health approach in Eastern Mediterranean countries

The One Health approach recognizes the interconnectedness of human, animal, and environmental health and promotes collaboration across various sectors to address complex health challenges, including AMR. In recent years, several Eastern Mediterranean countries have adopted the One Health approach to effectively combat AMR. *Egypt's National Action Plan*

In Egypt, the National Action Plan (NAP) for AMR was launched in 2017, integrating the human health, animal health, and environmental sectors within its framework [38]. The NAP focuses on several important areas. These include monitoring antimicrobial use and resistance patterns, improving infection prevention and control in healthcare settings, promoting responsible use of antimicrobials in both humans and animals, strengthening regulatory frameworks for AMS, and raising public awareness about the appropriate use of antibiotics. By implementing robust monitoring systems and data collection mechanisms, authorities can gather crucial information about AMR patterns and trends. This enables informed decision-making and targeted interventions to effectively combat resistance. The plan also emphasizes stewardship as an essential aspect. By promoting responsible antibiotic use across different sectors, such as healthcare facilities and livestock production units, Egypt aims to minimize unnecessary antibiotic consumption and reduce the development of resistant bacteria. Additionally, the NAP recognizes the pivotal role of infection prevention in curbing the spread of AMR. It emphasizes the importance of stringent infection control measures at healthcare facilities and the promotion of proper hygiene practices in various settings [39]. By taking significant steps towards an integrated approach involving human health, animal health, and environmental sectors, the Egyptian National Action Plan for Antimicrobial Resistance demonstrates the country's commitment to effectively mitigating the impact of AMR [39]. *Pakistan's National Action Plan*

Pakistan has also embraced the One Health approach to address AMR. In 2017, the country developed its NAP on AMR, incorporating the One Health concept [8]. The plan aims to strengthen AMR surveillance, promote rational use of antimicrobials, enhance infection prevention and control measures, and improve awareness and understanding of AMR among the general public and healthcare professionals [40]. *Challenges and Progress in Qatar*

While Qatar has successfully applied this approach for diseases like MERS-CoV, there are institutional and policy gaps [41]. A recent paper by Bansal et al. assessed the potential of the One Health Framework in Qatar from 2022-2027 [41]. Using Qatar's Joint External Evaluation (JEE) report and past experiences with zoonotic diseases, a multisectoral coordination outline was developed, and key recommendations were made, including improved communication, data-sharing policies, and greater resource allocation [41].

Challenges and opportunities

Despite the progress made in implementing interdisciplinary action and collaboration in the EMR, several challenges remain. Limited resources, fragmented health systems, knowledge gaps, and awareness are some of the key challenges faced by many countries in the EMR when it comes to combating AMR. Financial constraints often hinder the effective implementation of AMR containment strategies, leading to insufficient funding and a lack of human resources. This, in turn, may limit the capacity for surveillance, monitoring, and enforcement of regulations related to antimicrobial use. Furthermore, weak health systems and a lack of coordination among various sectors can impede the adoption of a One Health approach. It is crucial to strengthen health systems and enhance intersectoral collaboration for effective AMR containment. Additionally, limited awareness of AMR and its consequences among healthcare professionals and the public contributes to the inappropriate use of antimicrobials. Therefore, educational programs and public awareness campaigns are essential to change perceptions and promote responsible antimicrobial use. Opportunities for strengthening interdisciplinary action and collaboration in the EMR are abundant. Regional collaboration is crucial as cooperation among EMR countries can foster the sharing of knowledge, expertise, and best practices in tackling AMR. Joint initiatives and regional networks, such as the Eastern Mediterranean Antimicrobial Resistance Surveillance Network (EMARIS), can facilitate collaboration and the exchange of information. Furthermore, international partnerships with global organizations, such as the WHO, the Food and Agriculture Organization (FAO), and the World Organization for Animal Health (WOAH), can provide technical support and resources for implementing AMR containment strategies in the region. Lastly, research and innovation play a significant role in combating AMR. Fostering research and innovation in the field can help develop new diagnostic tools, vaccines, and alternative treatments. Collaborative research projects and partnerships between academia, industry, and governments can drive the development of novel solutions to combat AMR, ultimately bolstering interdisciplinary action and collaboration in the EMR (Table 1).

**Table 1:** Summary of the challenges and opportunities for combating antimicrobial resistance in the Eastern Mediterranean Region. AMR: Antimicrobial resistance; WHO: World Health Organization; OIE: World Organization for Animal Health; FAO: Food and Agriculture Organization

Aspect	Challenges	Opportunities
Resources	Limited financial resources leading to insufficient funding. Lack of human resources.	International partnerships with organizations like WHO, FAO, and OIE can provide technical support and resources. Joint initiatives and regional networks fostering collaboration and exchange of information.
Health Systems	Fragmented health systems. Lack of coordination among sectors impeding One Health approach adoption.	Strengthening health systems and enhancing intersectoral collaboration. Regional collaboration for sharing knowledge and best practices.
Awareness	Limited awareness of AMR among healthcare professionals and the public leading to inappropriate antimicrobial use.	Educational programs and public awareness campaigns to change perceptions and promote responsible use.
Research	Knowledge gaps.	Fostering research and innovation for new diagnostic tools, vaccines, and alternative treatments Collaborative research projects between academia, industry, and governments for novel solutions to combat AMR.
Collaboration	Limited regional collaboration.	Platforms like the Eastern Mediterranean Antimicrobial Resistance Surveillance Network (EMARIS) facilitating collaboration. Collaborative research projects fostering interdisciplinary action and collaboration in the EMR.

Future directions

The future of addressing AMR in the EMR relies on interdisciplinary collaboration and the adoption of the One Health approach. This concept recognizes the connections between human, animal, and environmental health. Since AMR does not adhere to geopolitical boundaries, effective containment requires joint efforts and synergies that bridge traditional disciplinary divides. The EMR, with its diverse healthcare infrastructure, economic capacities, and levels of public awareness, can greatly benefit from an interdisciplinary approach. Collaborative initiatives can help standardize methodologies, pool resources, and enhance knowledge sharing among countries, enabling them to combat AMR more efficiently and with a unified front. The One Health approach, due to its comprehensive perspective, provides an ideal framework for the EMR [42]. By acknowledging the shared environment and interconnected vulnerabilities of humans, animals, and ecosystems, the One Health approach encourages a multi-sectoral response to AMR. It prompts nations to focus not only on human medicine but also on veterinary and environmental practices, ensuring the judicious use of antimicrobials across all sectors. This comprehensive approach can significantly reduce the environmental reservoirs of resistance genes, preventing their transmission to human populations. Given the region's unique socio-political complexities and the challenges of war, migration, and varying health infrastructure, the One Health approach offers a unified strategy to optimize the health of all living beings and their shared environments. However, realizing the potential of interdisciplinary collaboration and the One Health approach in combating AMR in the Eastern Mediterranean requires more than just conceptual alignment. It necessitates strong political commitment, increased funding for AMR-related initiatives, and the strengthening of research and innovation capabilities. Nations must also prioritize public awareness campaigns to change perceptions and behaviors regarding antimicrobial use. Regional networks and collaborations, such as the Eastern Mediterranean Antimicrobial Resistance and Infection Surveillance Network (EMARIS), should be expanded to facilitate the exchange of knowledge and technical cooperation. By harnessing the opportunities inherent in interdisciplinary collaboration and the One Health framework, Eastern Mediterranean countries can pave the way for a future in which AMR is effectively managed, ensuring the health and well-being of their populations and ecosystems.

## Conclusions

AMR is becoming an increasingly serious threat to public health in Eastern Mediterranean countries. It is crucial to have a comprehensive and coordinated response to ensure effective AMS and contain the spread of AMR. This requires interdisciplinary engagement and cooperation, based on the One Health approach. Despite the challenges that exist, the region has already seen positive results through successful collaborative efforts, demonstrating the potential for further progress. To tackle AMR on a global level, it is essential to strengthen regional and international partnerships, enhance collaboration across sectors, and allocate resources to research and innovation.

## References

[1] Dhingra S, Rahman NA, Peile E (2020). Microbial resistance movements: an overview of global public health threats posed by antimicrobial resistance, and how best to counter. Front Public Health.

[2] Dadgostar P (2019). Antimicrobial resistance: implications and costs. Infect Drug Resist.

[3] Hernando-Amado S, Coque TM, Baquero F, Martínez JL (2019). Defining and combating antibiotic resistance from One Health and Global Health perspectives. Nat Microbiol.

[4] Balkhy HH, Zowawi HM, Alshamrani MM (2018). Antimicrobial resistance: a round table discussion on the "One Health" concept from the Gulf Cooperation Council Countries. Part Two: a focus on Human Health. J Infect Public Health.

[5] Lai CC, Chen SY, Ko WC, Hsueh PR (2021). Increased antimicrobial resistance during the COVID-19 pandemic. Int J Antimicrob Agents.

[6] Elmahi OK, Uakkas S, Olalekan BY (2022). Antimicrobial resistance and one health in the post COVID-19 era: what should health students learn?. Antimicrob Resist Infect Control.

[7] Talaat M, Tolba S, Abdou E, Sarhan M, Gomaa M, Hutin YJ (2022). Over-prescription and overuse of antimicrobials in the Eastern Mediterranean Region: the urgent need for Antimicrobial Stewardship Programs with access, watch, and reserve adoption. Antibiotics (Basel).

[8] Jirjees F, Ahmed M, Sayyar S, Amini M, Al-Obaidi H, Aldeyab MA (2022). Self-medication with antibiotics during COVID-19 in the Eastern Mediterranean region countries: a review. Antibiotics (Basel).

[9] Saleem Z, Hassali MA, Godman B (2020). Sale of WHO AWaRe groups antibiotics without a prescription in Pakistan: a simulated client study. J Pharm Policy Pract.

[10] Ahmadkhosravi N, Khosravi AD, Asareh Zadegan Dezfuli A, Hashemzadeh M, Saki M, Mehr FJ, Izadpour F (2021). Study of aerobic and anaerobic bacterial profile of nosocomial infections and their antibiotic resistance in a referral center, Southwest Iran: a three year cross-sectional study. PLoS One.

[11] Fahim NA (2021). Prevalence and antimicrobial susceptibility profile of multidrug-resistant bacteria among intensive care units patients at Ain Shams University Hospitals in Egypt-a retrospective study. J Egypt Public Health Assoc.

[12] Hasan R, Shakoor S, Baghdadi S, Mafi A, Aziz M (2016). Collaboration between tuberculosis control programs and the action plan for tackling antimicrobial resistance: an opportunity in the Eastern Mediterranean Region. Int J Mycobacteriol.

[13] Talaat M, Zayed B, Tolba S (2022). Increasing antimicrobial resistance in World Health Organization Eastern Mediterranean Region, 2017-2019. Emerg Infect Dis.

[14] Okeke IN, Laxminarayan R, Bhutta ZA (2005). Antimicrobial resistance in developing countries. Part I: recent trends and current status. Lancet Infect Dis.

[15] Borg MA, van de Sande-Bruinsma N, Scicluna E, de Kraker M, Tiemersma E, Monen J, Grundmann H (2008). Antimicrobial resistance in invasive strains of Escherichia coli from southern and eastern Mediterranean laboratories. Clin Microbiol Infect.

[16] Nellums LB, Thompson H, Holmes A (2018). Antimicrobial resistance among migrants in Europe: a systematic review and meta-analysis. Lancet Infect Dis.

[17] (2023). Global Database for Tracking Antimicrobial Resistance (AMR). http://www.amrcountryprogress.org/.

[18] Dyar OJ, Huttner B, Schouten J, Pulcini C (2017). What is antimicrobial stewardship?. Clin Microbiol Infect.

[19] Yusef D, Hayajneh WA, Bani Issa A (2021). Impact of an antimicrobial stewardship programme on reducing broad-spectrum antibiotic use and its effect on carbapenem-resistant Acinetobacter baumannii (CRAb) in hospitals in Jordan. J Antimicrob Chemother.

[20] Nassar H, Abu-Farha R, Barakat M, Alefishat E (2022). Antimicrobial Stewardship from Health Professionals' perspective: awareness, barriers, and level of implementation of the program. Antibiotics (Basel).

[21] Ababneh M, Issa N, Alkhatatbeh M (2017). Evaluation of core elements of antimicrobial stewardship programs in Jordanian hospitals. Jordan J Pharm Sci.

[22] Ababneh MA, Nasser SA, Rababa'h AM (2021). A systematic review of Antimicrobial Stewardship Program implementation in Middle Eastern countries. Int J Infect Dis.

[23] Saleh D, Abu-Farha R, Mukattash TL, Barakat M, Alefishat E (2021). Views of community pharmacists on antimicrobial resistance and Antimicrobial Stewardship in Jordan: a qualitative study. Antibiotics (Basel).

[24] Hassan SK, Dahmash EZ, Madi T (2023). Four years after the implementation of antimicrobial stewardship program in Jordan: evaluation of program's core elements. Front Public Health.

[25] Moradi G, Gouya MM, Eshrati B, Mohraz M, Molaei L, Piroozi B (2018). National action plan of the Islamic Republic of Iran for combating antimicrobial resistance during 2016 - 2021. Med J Islam Repub Iran.

[26] Alghamdi S, Shebl NA, Aslanpour Z, Shibl A, Berrou I (2018). Hospital adoption of antimicrobial stewardship programmes in Gulf Cooperation Council countries: a review of existing evidence. J Glob Antimicrob Resist.

[27] Hashad N, Perumal D, Stewart D, Tonna AP (2020). Mapping hospital antimicrobial stewardship programmes in the Gulf Cooperation Council states against international standards: a systematic review. J Hosp Infect.

[28] Al Salman J, Al Dabal L, Bassetti M (2021). Promoting cross-regional collaboration in antimicrobial stewardship: findings of an infectious diseases working group survey in Arab countries of the Middle East. J Infect Public Health.

[29] Alali WQ, AlFouzan W, Dhar R (2021). Prevalence of antimicrobial resistance in Gram-negative clinical isolates from a major secondary hospital in Kuwait: a retrospective descriptive study. Germs.

[30] OMAN SO: ANTIMICROBIAL RESISTANCE (AMR) NATIONAL ACTION PLAN (2024). WHO: Oman: Antimicrobial resistance (AMR) national action plan. https://www.who.int/publications/m/item/oman-antimicrobial-resistance-(amr)-national-action-plan.

[31] Nasr ZG, Jibril F, Elmekaty E, Sonallah H, Chahine EB, AlNajjar A (2021). Assessment of antimicrobial stewardship programs within governmental hospitals in Qatar: a SWOC analysis. Int J Pharm Pract.

[32] Alghamdi S (2024). University of Hertfordshire: The Adoption of Antimicrobial Stewardship Programmes in Ministry of Health Hospitals in Saudi Arabia. https://uhra.herts.ac.uk/handle/2299/21153.

[33] Alanazi M, Alqahtani HM, Alshammari MK (2023). Infection prevalence at a tertiary hospital in Hail, Saudi Arabia: a single-center study to identify strategies to improve antibiotic usage. Infect Drug Resist.

[34] Almansour K, Malik JA, Rashid I (2023). Physician's knowledge and attitudes on antibiotic prescribing and resistance: a cross-sectional study from Hail Region of Saudi Arabia. Healthcare (Basel).

[35] Alzahrani MA, Sadoma HH, Mathew S, Alghamdi S, Malik JA, Anwar S (2021). Retrospective analysis of antimicrobial susceptibility of uropathogens isolated from pediatric patients in tertiary hospital at Al-Baha Region, Saudi Arabia. Healthcare (Basel).

[36] El‐Lababidi RM, Mooty M, Bonilla MF (2019). Implementation and outcomes of an advanced antimicrobial stewardship program at a quaternary care hospital in the United Arab Emirates. J Am Coll Clini Pharm.

[37] Hamdan S, El‐Dahiyat F (2020). Implementation and evaluation of an antimicrobial stewardship program across nine hospitals in the United Arab Emirates: a qualitative study. J Pharm Pract Res.

[38] (2024). The American University in Cairo: The Egyptian National Action Plan of Antimicrobial Resistance: Governance Mechanism and Public Awareness.. Cairo.

[39] (2024). LSE: Developing National Strategies to tackle Antimicrobial Resistance across countries in the Eastern Europe, Middle East and Africa (EEMEA) region: A pilot analysis of Egypt, Russia and South Africa. https://globalcoalitiononaging.com/wp-content/uploads/2022/02/Developing-National-Strategies-to-tackle-Antimicrobial-Resistance-across-countries-in-the-EEMEA-region_13_10_Collated-All-Countries.pdf.

[40] Ahmed SK, Hussein S, Qurbani K, Ibrahim RH, Fareeq A, Ali Mahmood K, Mohamed MG (2024). Antimicrobial resistance: Impacts, challenges, and future prospects. J Med Surg Public Health.

[41] Bansal D, Jaffrey S, Al-Emadi NA (2023). A new One Health Framework in Qatar for future emerging and re-emerging zoonotic diseases preparedness and response. One Health.

[42] Nashwan AJ, Shah HH, Hussain T, Rauf SA, Ahmed SK (2024). Environmental drivers of antimicrobial resistance in low and middle-income countries: the impacts of a changing world. Environ Health Insights.

